# A logistic model for the detection of circulating tumour cells in human metastatic colorectal cancer

**DOI:** 10.1111/j.1582-4934.2012.01544.x

**Published:** 2012-09-26

**Authors:** Jorge Barbazán, María Vieito, Alicia Abalo, Lorena Alonso-Alconada, Laura Muinelo-Romay, Marta Alonso-Nocelo, Luís León, Sonia Candamio, Elena Gallardo, Urbano Anido, Andreas Doll, María los Ángeles Casares, Antonio Gómez-Tato, Miguel Abal, Rafael López-López

**Affiliations:** a Translational Laboratory Medical Oncology Department, Complexo Hospitalario Universitario de Santiago de Compostela/SERGASSantiago de Compostela, Spain; b Biomedical Research Unit, Research Institute Vall d'Hebron University HospitalBarcelona, Spain; c School of Mathematics, Universidad de Santiago (Campus Vida)Santiago de Compostela, Spain

**Keywords:** metastatic colorectal cancer, circulating tumour cells, biomarkers, GAPDH, VIL1, CD45, logistic model

## Abstract

The accuracy in the diagnosis of metastatic colorectal cancer (mCRC) represents one of the challenges in the clinical management of patients. The detection of circulating tumour cells (CTC) is becoming a promising alternative to current detection techniques, as it focuses on one of the players of the metastatic disease and it should provide with more specific and sensitive detection rates. Here, we describe an improved method of detection of CTC from mCRC patients by combining immune-enrichment, optimal purification of RNA from very low cell numbers, and the selection of accurate PCR probes. As a result, we obtained a logistic model that combines GAPDH and VIL1 normalized to CD45 rendering powerful results in the detection of CTC from mCRC patients (AUROC value 0.8599). We further demonstrated the utility of this model at the clinical setting, as a reliable prognosis tool to determine progression-free survival in mCRC patients. Overall, we developed a strategy that ameliorates the specificity and sensitivity in the detection of CTC, resulting in a robust and promising logistic model for the clinical management of metastatic colorectal cancer patients.

## Introduction

Colorectal cancer (CRC) remains the third most common and deadly cancer worldwide [1]. Improving diagnosis at early stages would represent a main achievement on progression-free and overall survival. Together with the progress of current diagnostic methods as colonoscopy, sigmoidoscopy and computerized tomography (CT) techniques, in the last years, quantification of CTC has excelled as a reliable, relatively inexpensive and minimally invasive detection method. Reverse transcriptase-polymerase chain reaction (RT-PCR), immunomagnetic isolation and enrichment, and microchip techniques have been developed to increase the accuracy and reliability of CTC detection [2]. Of note, the CellSearch System (Veridex LLC, Raritan, NJ, USA) that combines immunoenrichment and immunofluorescence for the detection of CTC received in 2007 Food and Drug Administration (FDA) clearance for the use as an aid in the monitoring of metastatic colorectal cancer. CTC quantification significantly correlated with progression-free survival (PFS) and overall survival (OS) after treatment or at baseline in metastatic CRC patients, arguing for the use of CTC as an independent prognostic factor that may be combined with others to improve assessment [3, 4]. Moreover, improvement of CTC detection and enumeration by combining immunoisolation and flow cytometry has led CTC to be proposed as a predictor of metastasis before the detection by conventional methods in CRC patients [5].

A principal challenge on CTC detection and enumeration is their rare frequency, ranging from one to hundreds of CTC among millions of other cell types, and therefore to move CTC evaluation forward into the clinical application, high levels of specificity and sensitivity are needed. Efforts have been made for the identification of RNA markers that offer increased detection rates when compared with multiparametric systems such as CellSearch [6]. Nevertheless, an effort in the development of more efficient techniques or in the convergence among different technical approaches is guaranteed to ameliorate the management of metastatic colorectal cancer patients.

In this work, we aimed to improve the accuracy of CTC detection by combining immunoisolation and enrichment of CTC with cDNA pre-amplification and RTqPCR quantification specific transcripts. The result is a combination of three biomarkers that effectively detects CTC in metastatic CRC patients in terms of high specificity and sensitivity. This strategy that synergized an accurate technical approach with the use of potent biomarkers allowed us to evaluate the impact of CTC on patient outcome.

## Materials and methods

A total of 44 metastatic colorectal cancer patients were enrolled between June 2009 and October 2010 at Complexo Hospitalario Universitario de Santiago, Santiago de Compostela (Spain). Informed consent approved by the ethical committee was signed by all patients. Inclusion criteria were the presence of measurable metastatic colorectal cancer (stage IV), an Eastern Cooperative Oncology Group (ECOG) performance status not greater than 2 and the starting of a systemic chemotherapy based on fluoropirimidines with or without oxaliplatin/irinotecan and therapeutic antibodies. Disease progression, evaluated by computerized tomography, was defined following RECIST 1.1 guidelines [7] as an increase in the number of metastatic lesions, growth of existing lesions in more than 20% or both during treatment. Furthermore, 22 healthy individuals with similar age ranges to those of patients were included as negative controls (Table 1).

**Table 1:** Patients and clinicopathological data

Age (years)		
Mean	64.7	
SD	9.6	
Range	42–83	
Gender	*N*	%
Male	33	75
Female	11	25
Primary tumour location		
Colon	33	75
Rectum	10	22.7
Both	1	2.3
K-ras status		
WildType	22	50
Mutated	14	31.8
Unknown	8	18.2
pT		
pT_1_-pT_2_	1	2.3
pT_3_	25	56.8
pT_4_	10	22.7
pT_X_	8	18.2
pN		
pN_0_	4	9.1
pN_1_	13	29.5
pN_2_	15	34.1
pN_X_	12	27.3
Number of metastatic sites		
1	24	54.5
≥2	20	45.5
Metastasis location		
Liver	19	43.2
Liver and other	17	38.6
Non liver	8	18.2
Therapy scheme		
Folfox	31	70.5
Folfiri	7	15.9
Capecitabine	5	11.4
Irinotecan	1	2.3

### Colorectal cancer cell lines

Colorectal carcinoma HT29 and HCT116 cell lines were maintained in McCoy's 5A medium (Gibco, Invitrogen, Carlsbad, CA, USA) supplemented with 10% foetal bovine serum and 1% penicillin-streptomycin. SW480 cell line was maintained in RPMI medium (Sigma-Aldrich, St. Louis, MO, USA), and supplemented with 10% FBS, 1% penicillin-streptomycin and 5 mM l-Glutamine.

### CTC isolation and quantification

Circulating tumour cells were isolated and enriched from 7.5 ml of peripheral blood from patients by using the CELLection™ Epithelial Enrich system (Invitrogen, Dynal, Oslo, Norway). Beads are coated with a monoclonal antibody towards the human epithelial cell adhesion molecule (EpCAM), highly expressed in cells of epithelial origin. Circulating Tumour Cells (CTC) were isolated following manufacturer's instructions with little modifications. Briefly, we mixed 7.5 ml of blood with the recommended buffer, centrifuged at 1250 × *g* for 15 min and discarded the plasma fraction supernatant. The pellet was mixed again with buffer and 100 μl of beads was added. After incubation for 30 min. at 4°C in continuous rotation, magnetic bead-bound CTC were washed three times with the help of a magnet and directly resuspended in 100 μl of RNAlater® solution (Ambion, Austin, TX, USA) and stored at −80°C. Total RNA from CTC was extracted in the presence of a polyRiboA as a carrier with the Qiamp Viral kit (Qiagen, Valencia, CA, USA), optimized for very low cellularity samples. cDNA was synthesized by using Superscript III chemistry (Invitrogen) with a final RNAseH (Invitrogen) treatment. To maximize detection rates, we performed a pre-amplification step by using the TaqMan® PreAmp Master Mix kit from Applied Biosystems with 14 reaction cycles. Pre-amplificated products were subjected to TaqMan® real-time PCR amplifications (RTqPCR). This optimization for CTC detection provided an increased sensitivity of 6-7 Ct's, threshold cycle as a relative measure of the concentration of target in a PCR reaction, without compromising the reliability of the original RNA proportions (data not shown). The same extraction protocol was applied to healthy volunteer's blood, to evaluate the levels of unspecifically haematopoietic isolated cells.

In parallel and to ameliorate the specificity of CTC detection, RTqPCR probes were selected by a demonstrated high expression in organs of the digestive tract, low or null expression in those cells with haematopoietic origin, and high and sustained expression in colorectal carcinomas, from the Human Protein Atlas database. Genes fitting these criteria that were included in this study were Villin 1 (VIL1), Cytokeratin 20 (CK20), Tetraspanin-8 (TM4SF3), EpCAM, Family with sequence similarity member A (FAM132A), Cadherin 17 (CDH17), T-box transcription factor 20 (TBX20), Caudal type Homeobox trascription factor 2 (CDX2), Cell surface A33 antigen (GPA33) and Phosphatidylinositol-3,4,5-trisphosphate-dependent Rac exchange factor 2 (PREX2). We further included Glyceraldehyde-3-phosphate dehydrogenase (GAPDH) as a housekeeping gene for the evaluation of the total number of isolated cells (specific CTC plus unspecific haematopoietic cells), and Protein tyrosine phosphatase, receptor type C (CD45) as a specific marker for cells with a haematopoietic origin and used for the estimation of unspecificity during CTC immunoisolation.

### Statistical analysis

Data were analysed with the R package and SPSS (Chicago, version 15.00 for Windows). Statistical differences were considered with a *P*-value lower than 0.05.

## Results

### Epithelial cell markers efficiently detect CTC in stage IV CRC patients

**Fig 1:**
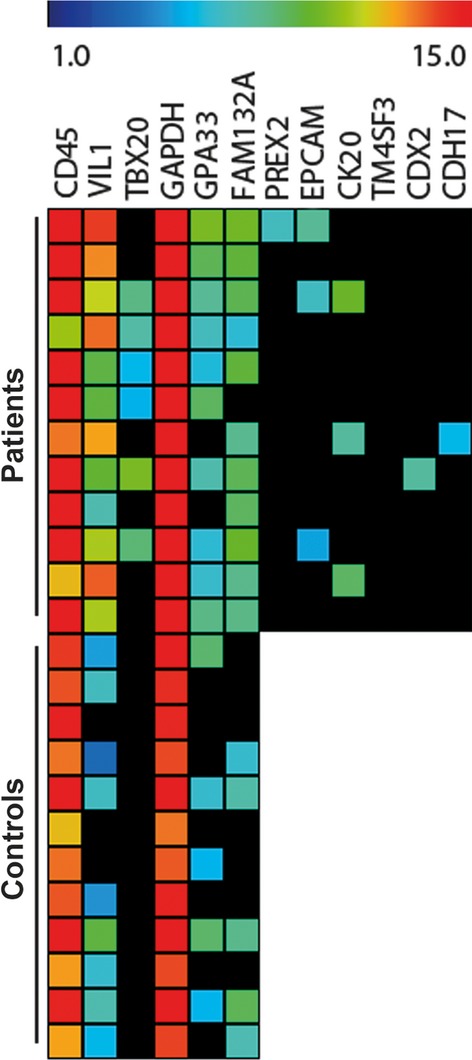
Heatmap representing the sensitivity and specificity of selected probes. Probes were tested into a series of twelve stage IV CRC patients. Only six of the selected twelve probes were able to detect CTC in at least 50% of patients (CD45, VIL1, TBX20, GAPDH, GPA33 and FAM132A). Those six probes were selected for further studies and evaluated in twelve control samples from healthy volunteers for specificity analysis. Strong signals were detected for GAPDH and CD45 as expected, VIL1, GPA33 and FAM132A showed a variable degree of specificity and no signal was detected in controls for TBX20. Colour scale shows higher expression for these probes in patient samples compared with controls.

Taqman probes targeting those genes selected from database as highly and specifically expressed in colorectal carcinomas (see Materials and methods) were first screened for their capability to detect CTC within the first twelve samples from metastatic colorectal patients. Those probes presenting signal in at less 50% of the samples when analysed by RTqPCR included VIL1, FAM132A, TBX20 and GPA33 as intestinal tissue-specific probes, and GAPDH and CD45 as expected for a housekeeping gene and a marker for unspecific cell content, respectively (Fig. 1; Patients group). Of note, the disparity of these results with the percentage of CK20 positive patients upon CTC immunoisolation in stage IV CRC patients (77.2%; [8]) could be explained by the different technique employed for the detection. The next step consisted of the evaluation of the positive probes in a cohort of twelve control samples from non-affected volunteers. Equal amounts of blood (7.5 ml) obtained from age-matched controls were processed as those from the group of patients to assess the specificity of the selected candidate biomarkers. GAPDH and CD45 were positive as predicted, while only TBX20 presented negative values in the group of controls among the four specific intestinal probes, suggesting a basal expression in lymphocytes for VIL1, GPA33 and FAM132A (Fig. 1; Controls group). Of note, the heatmap representing the intensity of expression for the selected genes showed that either the number of positive samples or the intensity of expression was lower in the group of controls compared with patients, with the exception of CD45 as a marker of unspecificity (Fig. 1). To complete the analysis of specificity and sensitivity of the selected probes, we analysed their expression in the colon cancer cell lines HT29, HCT116 and SW480. All probes were positive in all cell lines, except for CD45 that showed null expression and confirmed its accuracy as a marker of unspecific non-CTC immunoisolation (data not shown).

**Fig 2:**
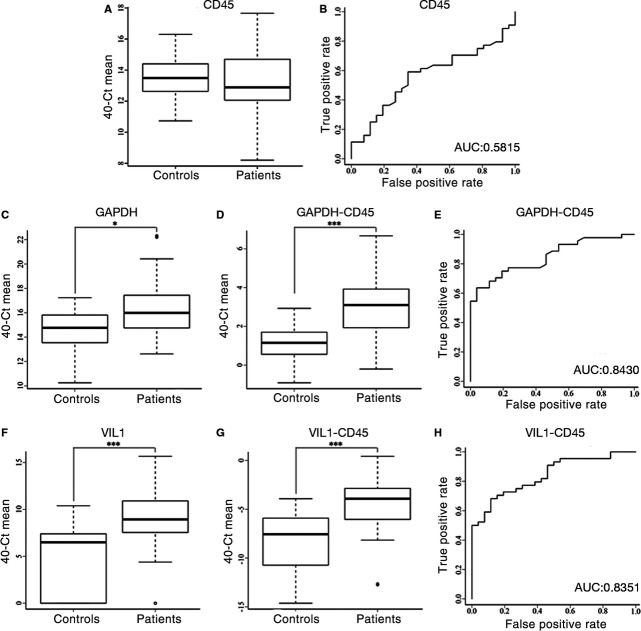
Accuracy of GAPDH and VIL1 normalized to CD45 in CTC detection. Box plots indicate median values in the group of control compared with the group of metastatic CRC patients for CD45 (A), GAPDH (C), GAPDH normalized to CD45 (D), VIL1 (F) and VIL1 normalized to CD45 (G). Of note, while CD45 showed no differences between both groups, GAPDH and VIL1 demonstrated optimal accuracy in the detection of CTC from metastatic CRC patients when normalized to CD45 as a marker of unspecific non-CTC cells (*P* < 0.001). This was further demonstrated when evaluated the specificity and sensitivity of these probes with the AUROC values. As expected, CD45 demonstrated no utility to discriminate between both groups of samples (B), while GAPDH (E) and VIL1 (H) normalized to CD45 showed promising AUROC values for the detection of CTC from metastatic CRC patients.

We next evaluated the quality of these candidates as biomarkers for the detection of CTC by comparing the group of metastatic colorectal cancer patients with the group of controls. CD45 presented similar median values, indicating that the degree of unspecificity in the isolation of CTC was the same for both groups (*P* = 0.2613; Fig. 2A). In contrast, GAPDH showed significant increased levels in the group of CRC patients when compared with controls (*P* = 0.01022; Fig. 2C), confirming that in addition to the unspecific isolation of lymphocytes, the CELLection method positively isolates epithelial CTC. Furthermore, when GAPDH levels were normalized to CD45 values, thus representing the CTC after subtracting the unspecific non-CTC contribution to total GAPDH, the statistical significance highly increased (*P* = 0.000002286; Fig. 2D). Concerning the four intestinal-specific probes, only VIL1 presented statistically significant differences among the group of CRC patients and controls (*P* = 0.00015; Fig. 2F); although FAM132A, GPA33 and TBX20 presented a reasonable specificity, they failed to demonstrate an acceptable sensitivity for the detection of colorectal CTC (data not shown). The normalization of VIL1 signal to CD45 values also increased the significance of this biomarker (*P* = 0.000003856; Fig. 2G), although this benefit was not as notable as with GAPDH. This is consistent with VIL1 being a specific biomarker for intestinal epithelial tissue.

Once we validated GAPDH and VIL1 as reliable individual biomarkers, we analysed their diagnostic potential by performing an analysis of Receiver Operating Characteristic (ROC) curves and the area under the curves or AUROC (Area Under ROC curves). Using this approach, we obtained AUROC values of 0.5815 for CD45 (Fig. 2B), as might correspond for an unspecific marker, while 0.8430 for GAPDH (Fig. 2E), and 0.8351 for VIL1 (Fig. 2H), further validating both genes as reliable markers for the detection of CTC in metastatic CRC patients.

### The combination of VIL1, GAPDH and CD45 generates a potent logistic model for the detection of CTC in colorectal cancer

**Fig 3:**
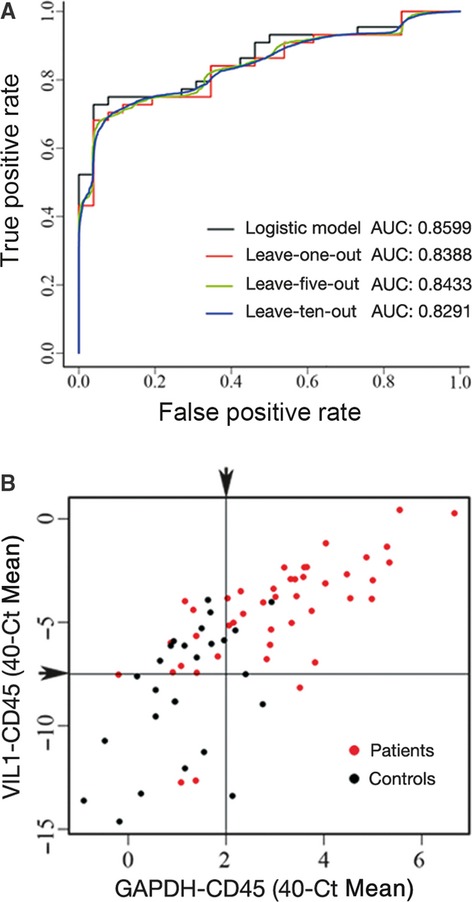
Logistic model for the detection of CTC from metastatic CRC patients. (A) The combination of GAPDH and VIL1 normalized to CD45 by multivariate analysis rendered a logistic model that improved the accuracy of the individual probes as demonstrated by AUROC analysis (0.8599). The robustness of the model was further confirmed through a cross-validation strategy, demonstrating acceptable behaviour when leaving one, five or even ten samples out of the model. (B) Dot-plot representing sample distribution [patients (red dots) and controls (black dots)]. Cut-off values were established in 2 for GAPDH normalized to CD45 and −7.5 for VIL1 normalized to CD45.

We next sought to evaluate whether a combination of probes could ameliorate the sensitivity and specificity of the detection of CTC compared with individual biomarkers. For this, we performed a multivariate analysis by using binary logistic regression with GAPDH, CD45, VIL1, TBX20, FAM132A and GPA33 levels. This analysis generated a model including VIL1, GAPDH and CD45, which is consistent with the data presented above concerning the robustness of the biomarkers. As mentioned, CD45 was not able to discriminate between control and metastatic CRC groups, but demonstrated utility as a normalizing gene to improve the discriminating power of GAPDH and VIL1. Of note, CD45 presented a negative coefficient in the model, while it was positive for GAPDH and VIL1, thus the former normalizing the later biomarkers for a better performance of GAPDH and VIL1 in the detection of CTC in metastatic CRC patients. The diagnostic efficacy of this model was assessed with ROC analysis, obtaining an AUROC of 0.8599 (Fig. 3A). These results reinforced GAPDH and VIL1 normalized to CD45 as accurate biomarkers for the detection of CTC from metastatic colorectal cancer patients, and further indicate their combination as a more efficient detection method than GAPDH or VIL1 biomarkers individually. Finally, we used a cross-validation strategy to assess the reliability of the logistic model using the combination of GAPDH, VIL1 and CD45 as the best model for colorectal CTC detection. We observed an acceptable behaviour of the model by the ‘leave-one-out’ statistic cross validation (AUROC 0.8388; Fig. 3A), as well as when we used the “leave-five-out” statistic (AUROC 0.8433; Fig. 3A), or the ‘leave-ten-out’ validation (AUROC 0.8291; Fig. 3A).

The analysis of the distribution of patients and controls based on the levels of GAPDH and VIL1 normalized to CD45 resulted in a clear separation of both populations (see areas depicted in Fig. 3B). Focusing on the overlapping zone, we obtained an optimal cutting point of GAPDH-CD45 = 2 (40-Ct Mean) and VIL1-CD45 = −7.5 (40-Ct Mean), rendering 72.7% of true positive metastatic CRC patients with a 9.09% of false positives (Fig. 3B). All these data demonstrated that the model of GAPDH, VIL1 and CD45 biomarker combination is a robust method for the detection of CTC from metastatic colorectal cancer patients.

### The logistic model including GAPDH, VIL1 and CD45 correlates with progression-free survival

**Fig 4:**
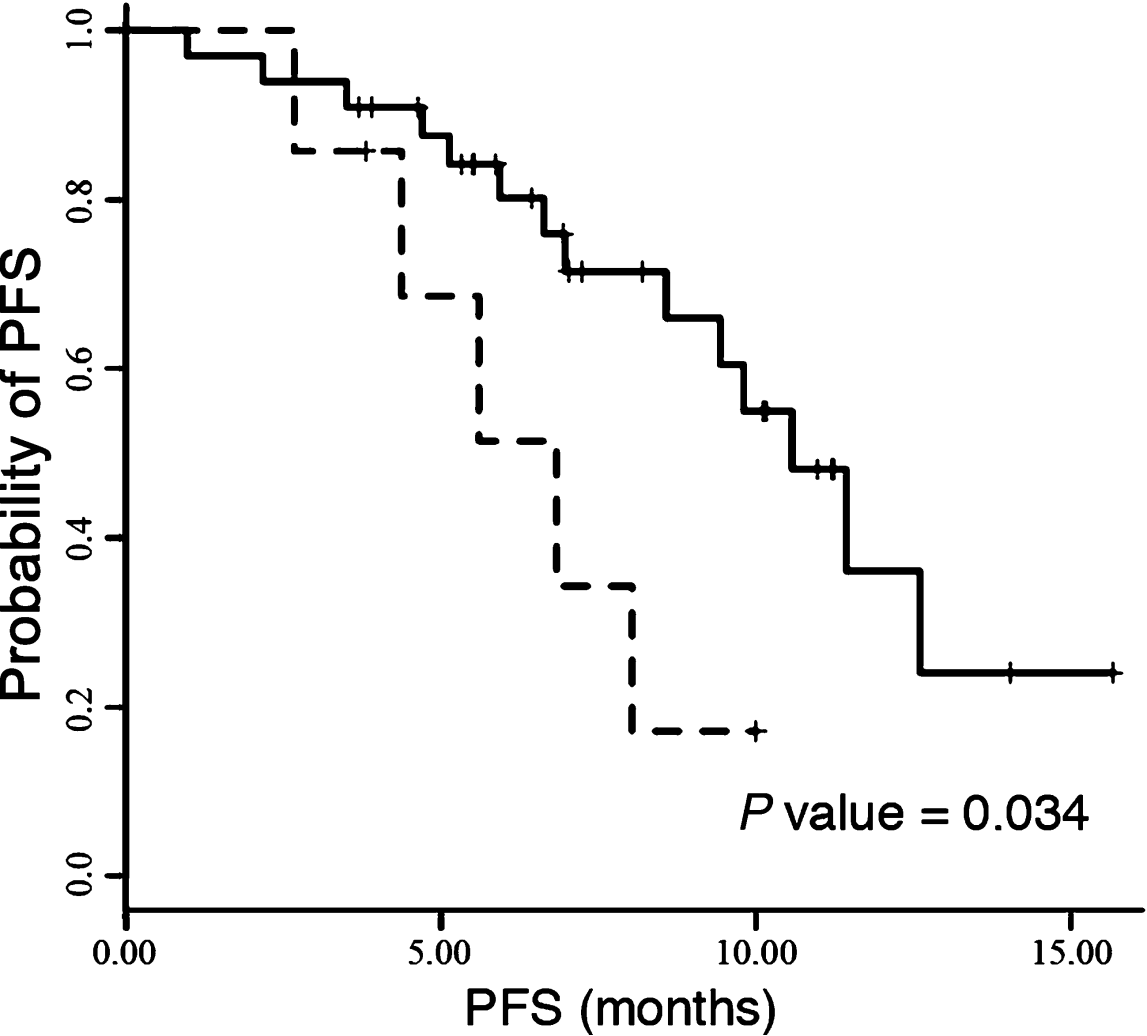
Performance of the logistic model in the prognosis of metastatic CRC. Kaplan–Meier analysis demonstrating the accuracy of the logistic model including GAPDH and VIL1 normalized to CD45 at the clinical setting. PFS of metastatic CRC patients with CTC below the cut-off value of 4.5 for GAPDH normalized to CD45 and -2 for VIL1 normalized to CD45, was significantly better (continuous line), compared with metastatic CRC patients with CTC values above the cut-off (dashed line) (*P* < 0.05).

Once we improved the sensitivity and specificity of the method of CTC detection, we sought to evaluate the validity of the logistic model at the clinical setting. For this, we analysed the time to progression in the group of metastatic CRC patients included in the study related to the quantification of CTC with the logistic model including GAPDH and VIL1 normalized to CD45. For this, we selected a subset of 40 evaluable patients. Progression-free survival was defined as the elapsed time between the onset of a first-line therapy and disease radiological progression or patient death. As shown in Figure 4, the probability of a patient to progress was directly correlated with the levels of CTC measured by the logistic model; Kaplan–Meier analysis of metastatic CRC patients with cut-off values of -2 (40-Ct mean) for VIL1-CD45, and 4.5 (40-Ct mean) for GAPDH-CD45, rendered significantly different PFS (*P* < 0.05; Fig. 4). Patients with CTC marker levels higher than cut-off values presented a median PFS of 6.8 months, whereas in those patients with marker levels below cut-off, PFS times increased up to 9.8 months (*P* < 0.05; Fig. 4). These results validate the logistic model including GAPDH, VIL1 and CD45 as a reliable method for the analysis of CTC and as an accurate predictor of time to progression in metastatic CRC patients.

## Discussion

We present here the optimization of CTC immunoisolation and quantification with two main focuses: first, the improvement of sensitivity on the detection technology and second, the selection of appropriate probes looking for a higher specificity. To increase the sensitivity of the detection, we adapted the method originally described by Rigau and co-workers for the detection of prostate cancer in urine [9], to the immunoisolation of CTC from metastatic colorectal cancer patients.

Antibody-based enrichment coupled to magnetic particles represents an accurate method for CTC isolation, widely accepted at the clinical setting as stated by the American FDA approval for breast, colorectal and prostate cancer. Compared with other methods of immunobead isolation, the positive enrichment protocol based on antibodies recognizing the EpCAM has shown in our hands a better performance in CTC isolation (data not shown). Antibody-based enrichment strategies, however, are limited by the fact that CTC with absent or low target antigen expression may be missed by these methods. A combination of cell-surface antibodies can be required for improved recovery rates of CTC in a number of types and subtypes of tumours due to variable expression levels of a particular biomarker [10]. In addition, the expression of EpCAM might be modulated during the process of epithelial-to-mesenchymal transition that is closely related to the initial steps of tumour cell dissemination, with the subsequent impact on CTC isolation [11]. Nevertheless, CTC enrichment with EpCAM-coupled antibodies has demonstrated to be superior to other cytometric methods and to be a reliable method for CTC detection in metastatic CRC patients [12]. Likewise, colorectal carcinomas present a high and constant expression of EpCAM, reaching recovery rates of up to 80% and making EpCAM-based immunoenrichment the method of choice [13]. Importantly, EpCAM is apparently needed to maintain distinct cancer cell attributes and, potentially, the cancer stem-cell phenotype [14]. CD133+ cells, currently one of the best markers to characterize colon cancer stem cells and an independent prognostic marker that correlates with low survival, are positive for EpCAM [15]. Finally, antibodies against EpCAM can efficiently target colorectal tumour-initiating cells [16], conferring a considerable value to the EpCAM-isolated CTC population in terms of therapeutic intervention.

Circulating tumour cells enrichment coupled to RNA purification using a kit specifically designed for low abundance samples plus pre-amplification before proceeding to real-time quantitative PCR provided with optimal detection rates. Once we improved the sensitivity of the method, we sought to ameliorate its specificity by selecting probes with a concrete pattern of expression, specific for the epithelial cells originated from the colorectal carcinoma and not expressed in other cells types present in the blood (*i.e*. white cells). After screening all selected probes, GAPDH as a marker of global cellularity and VIL1 as a marker of intestinal epithelial origin, both normalized to CD45 as a marker of unspecific non-CTC immunoisolation, were included in a statistical model that efficiently discriminated between metastatic colorectal cancer patients and controls. Compared with other techniques aiming to detect CTC upon isolation and enrichment, our CTC detection methodology developed for metastatic CRC patients demonstrated to be superior to CK20 RT-PCR (AUROC: 0.672; [17]), or to the CellSearch system when the group of metastatic patients was compared with localized disease (AUROC: 0.79; [18]).

Likewise, our results demonstrated that the model combining GAPDH and VIL1 normalized to CD45 as biomarkers of CTC has prognostic significance in metastatic colorectal cancer, in agreement with previous studies [3, 19], and a number of molecular biomarkers demonstrating clinical utility in determining prognosis [8, 20]. The prognostic value of CTC must be related to the burden of the systemic disease. Although the number of patients in our study was limited and a large study is guaranteed, there remained prognostic significance either when we grouped the patients in disease progression due to an increase in the number of metastatic lesions (*P* < 0.05), or when they progressed due to an augmentation in the size of the lesions (*P* < 0.05). These results suggest that whether the CTC reflect the risk of generating new metastatic lesions or the consequence of the delivery of tumour cells into the bloodstream associated with an actively growing lesion, elevated levels of CTC correlate with the global extension of metastatic disease.

At the therapeutic levels, CTC have shown to be promising as a predictor of resistance to chemotherapy [21] to guide therapy choice and to aid in patient selection for targeted agents [22]. Furthermore, it has been suggested that more aggressive CTC share genotypic characteristics with cancer stem cells, characterized by multidrug resistance, which allow these cells to drive tumour growth evading apoptosis and conventional therapy [2]. The optimal behaviour demonstrated by our technique and biomarkers in the detection of CTC from metastatic colorectal cancer patients might provide utility in the detection of CTC for monitoring of therapeutic response in metastatic CRC patients. Regarding our study, with the vast majority of patients treated with a therapeutic scheme based on 5-Fluorouracil, we are currently applying this logistic model in the therapeutic follow-up of our patients cohort. To date, the CTC count before and during treatment has proven to independently predict PFS and OS in advanced colorectal cancer patients treated with chemotherapy plus targeted agents [23]. It represents a challenge to efficiently monitor the response of advanced cancer patients to therapy, and a significant improvement in the sensitivity and specificity of CTC detection is a realistic strategy to really impact in the management of the disease.
